# Supplementary material for: Background proteome correction promotes confident identification of dynamic protein-protein interactions between different biological contexts
Source: bioRxiv. 2026 Sep 2:2026.09.01.748607. Preprint. [Version 1] doi: 10.64898/2026.09.01.748607 (PMC13684606; doi:10.64898/2026.09.01.748607)
Supplement: Supplementary file 1 [file NIHPP2026.09.01.748607V1-supplement-1.pdf]

bioRxiv preprint doi: https://doi.org/10.64898/2026.09.01.748607; this version posted September 3, 2026. The copyright holder for this preprint (which was not certified by peer review) is the author/funder, who has granted bioRxiv a license to display the preprint in perpetuity. It is made available under a CC-BY 4.0 International license.

Supplementary Data

Supplementary Figure 1.

A

Homology arm (HA)	Sequence
5' HA	tgctggctcttgccccatagggagcccttaacccggagttgcctagacagggttgtcgcatgcgctctctacagtcatgtttgtcgcaatggaccttgggcggaaggtta<br>cgtaagagcaaatgactttgattacagtcacttggtcggtagtcttgttacgcgctacctccgcggggtacttgattataacagaaagtagttccggcttgtGTTGTTT<br>CGGTCGAGGAGCCCTCGCCGCCATTTCAAGACAGTGAGAGGTAGATGTCTACCAAGAGTTCCTAAGATTCGAGTT<br>CAGTATTCTGTGCCGCCCCCTGAGCCCTTCAAAACACTGATACGGCTGCTGCCTTTTTTCTGCTACTTCTAGAACC<br>ATTCTTGTCAGTAGTGGCGGCAGTAGAAGTCAATTACCCCTTGTTCTATCTCAAGAAGCTCCAG
3' HA	GCGTCTTCCGTGGGCAACGTGGCCGACAGTACAGgtactagtgtcccattctactctggcagacgtccctttggggtctggcgccgtcttgtatcttt<br>cattacttccctctgaccatccccattacatcagtggcattactgagctctcgagttctactgctatctgatcgggtttggtgctgccgtccgttcgtaaacattctctagttgcttg<br>cttgcattttgcttgctttgggggctttttcgttgttgctttatcccatcagtgttgtagccttctctctcgggacgaatctatagcacacgtgcgcgcttagtgtgtcttggaatggg<br>ctgcattggaatctctgcaatttctgtctcccgtcgcaactgttttctccctcaatttcagcccgtcgctccctcccgtgcgcttccacctcctccccaaagcccctttcccttagc<br>cacatttggtcctccttgccaggctagggcggggatgctatttcggtgtaatcctagcctcactttgcatcaggcatgactagcgaaggctcctcggtgtgcttccaagta<br>gcaccaagaagagctacttgagcccgcaccccgccggggcagcgccttgtgttcttgggttgagcggccccgatttaggttggaaatttccaggggttctcttagccat<br>cacccaattgcacccgctgccccagtacggagtttcctaccctggtcagccctccctgcttgttgcaaccacacccttcctctgcgaacgtgccagcccgacagtcgttt<br>cttatcctcagggaagaaaccacctcggtgtgttgcttggcggcgaccaccctgcccgccctcctaattctctttctttgtgttactttctctatgtattcctccgccatggcctct<br>gggttttcatccccattctttagcccagtatctctttatccatagggtgtgcattattatttctttttaagtttccatccttgcttgaagagtttgtaaagcaactgg

B

Primer	Sequence
Amplifies 5' HA with homology to pDONR plasmid (F)	cgttatcccctgattctgcaccgggtcttcTGCTGGCTCTTGCCCCATAG
Amplifies 5' HA with homology to PuroR-Flag gBlock (R)	gcagccgtatcagtgttttgaaGGGC
Amplifies PuroR-Flag gBlock with homology to 5' HA (F)	tcaaaacactgatacggctgctgcc
Amplifies PuroR-Flag gBlock with homology to 3' HA (R)	ATGGTCAGAGGGAAGTAATGAAAGATACAAGAC
Amplifies 3' HA with homology to PuroR-Flag gBlock (F)	tcttgtatctttcattacttccctctgaccatcc
Amplifies 3' HA with homology to pDONR plasmid (R)	gcggtaatacggttatccaaggtcttcCCAGTTGCTTTACAAACTCTTC
Genotyping primer (F) outside of 5' HA	gcccttccgccttgctcttaaaaac
Genotyping primer (R) outside of 3' HA	CCG CCC CAA ACA GCC TCA ATC T

Figure S1A. Table containing sequences of the 5' and 3' homology arms (HAs) added to the repair template for knock-in into the endogenous *Ogt* locus.

Figure S1B. Table containing primer sequences used to clone the *Ogt* repair template (HAs, PuroR-T2A-3xFLAG or 2x-Strep cassette) into a pDONR-BFP plasmid.

bioRxiv preprint doi: https://doi.org/10.64898/2026.09.01.748607; this version posted September 3, 2026. The copyright holder for this preprint (which was not certified by peer review) is the author/funder, who has granted bioRxiv a license to display the preprint in perpetuity. It is made available under a CC-BY 4.0 International license.

Supplementary Figure 2.

A

Interactor	Confirmed OGT interaction?	Method of validation	Source(s)
HCF-1	Yes	Reciprocal IP-MS	Wysocka et al. *Genes & Dev*, 2003
TET2	Yes	Reciprocal IP-western blot	Vella et al. *Mol Cell*, 2013
Sin3A	Yes	Reciprocal IP-western blot	Yang et al. *Cell*, 2002
PROSER1	Yes	Reciprocal IP-western blot	Wang et al. *Life Sci Alliance*, 2022
SETD1A	Yes	Reciprocal IP-MS	Deplus et al. *EMBO J*, 2012
BAP1	Yes	Reciprocal IP-MS	Dong et al. PNAS, 2025

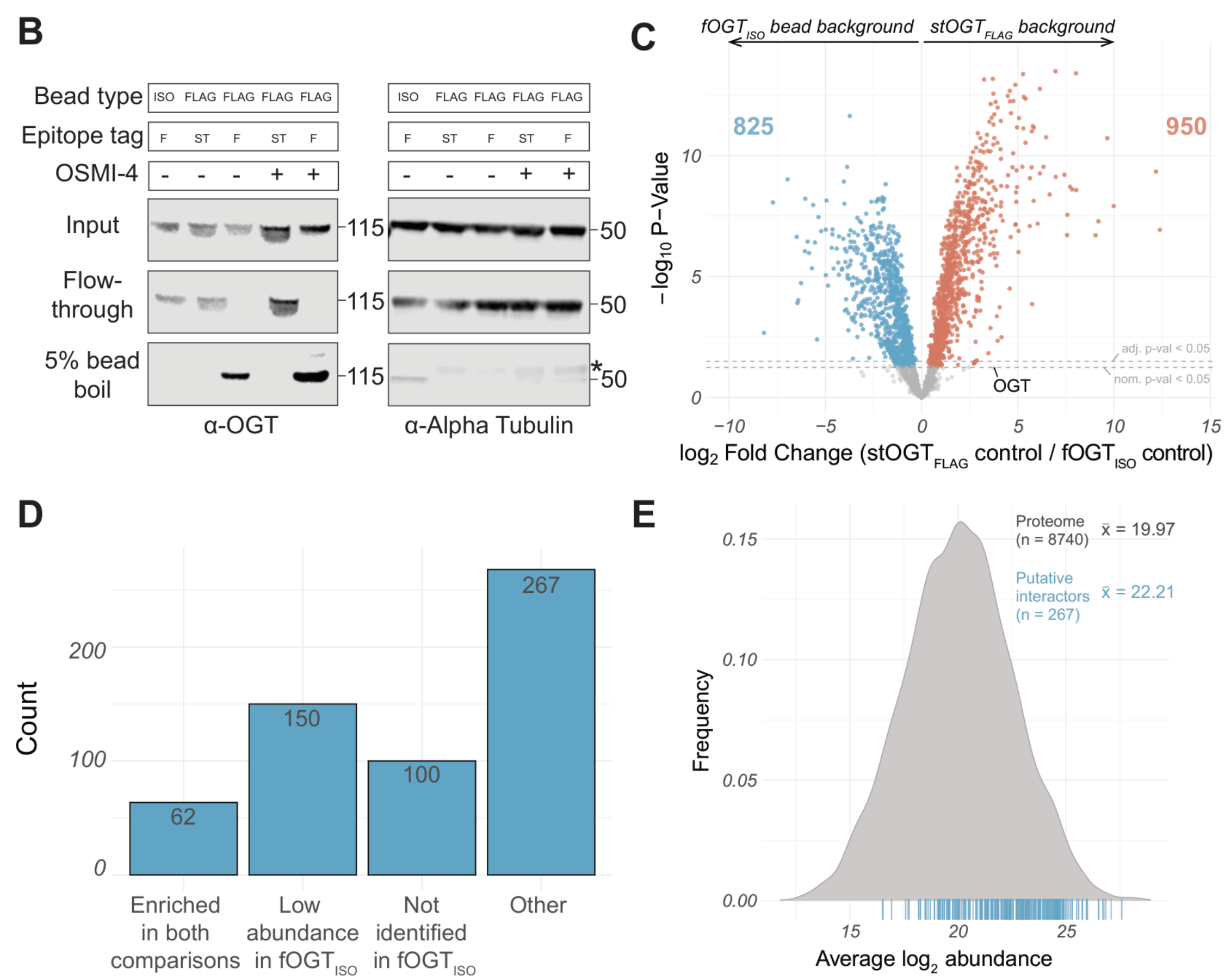

Figure S2A. Table containing validated OGT interactors. A protein is a validated OGT interactor if it has been identified to interact with OGT through some orthogonal method (to OGT co-IP-MS), such as reciprocal immunoprecipitation.

Figure S2B. Western blot depicting anti-FLAG OGT enrichment efficiency. Input, flow-through/unbound, and supernatant from 5% boiled beads (eluate) were probed with anti-OGT and anti-Alpha Tubulin antibodies to show FLAG-specific depletion of OGT from mESC lysate and presence on anti-FLAG beads. Asterisk indicates antibody heavy chain.

Figure S2C. Volcano plot comparing the fOGT$_{ISO}$ and stOGT$_{FLAG}$ controls, an ancillary comparison as an example. Orange points are proteins specifically enriched in stOGT$_{FLAG}$

bioRxiv preprint doi: https://doi.org/10.64898/2026.09.01.748607; this version posted September 3, 2026. The copyright holder for this preprint (which was not certified by peer review) is the author/funder, who has granted bioRxiv a license to display the preprint in perpetuity. It is made available under a CC-BY 4.0 International license.

samples, and light blue points are proteins specifically enriched in $fOGT_{ISO}$ samples. Gray points have nominal $p > 0.05$. Bolded values specify proteins passing the nominal p-value threshold $p < 0.05$.

Figure S2D. Bar graph categorizing the 579 putative interactors identified in the $fOGT_{FLAG}$ vs. $fOGT_{ISO}$ comparison. "Enriched in both comparisons": $log_2FC > 0$ & $p < 0.05$ in the $fOGT_{FLAG}$ vs. $fOGT_{FLAG}$ comparison and $fOGT_{FLAG}$ vs. $stOGT_{FLAG}$ comparisons. "Low abundance": bottom 20% of abundance in $fOGT_{ISO}$ samples. "Not identified": proteins not detected in $fOGT_{ISO}$ samples. "Other" encapsulates all leftover putative interactors.

Figure S2E. Density plot demonstrating abundance bias of "other" putative interactors. Log_2 average abundance of proteins in the 3x-FLAG-OGT mESC proteome are plotted. The 274 "Other" proteins from **Fig. S2D** are plotted via a light blue rug plot below the density plot. The x-bar values in the top right corner detail the mean abundance of proteins across the proteome and the mean abundance of the "other" putative interactors.

bioRxiv preprint doi: https://doi.org/10.64898/2026.09.01.748607; this version posted September 3, 2026. The copyright holder for this preprint (which was not certified by peer review) is the author/funder, who has granted bioRxiv a license to display the preprint in perpetuity. It is made available under a CC-BY 4.0 International license.

Supplementary Figure 3.

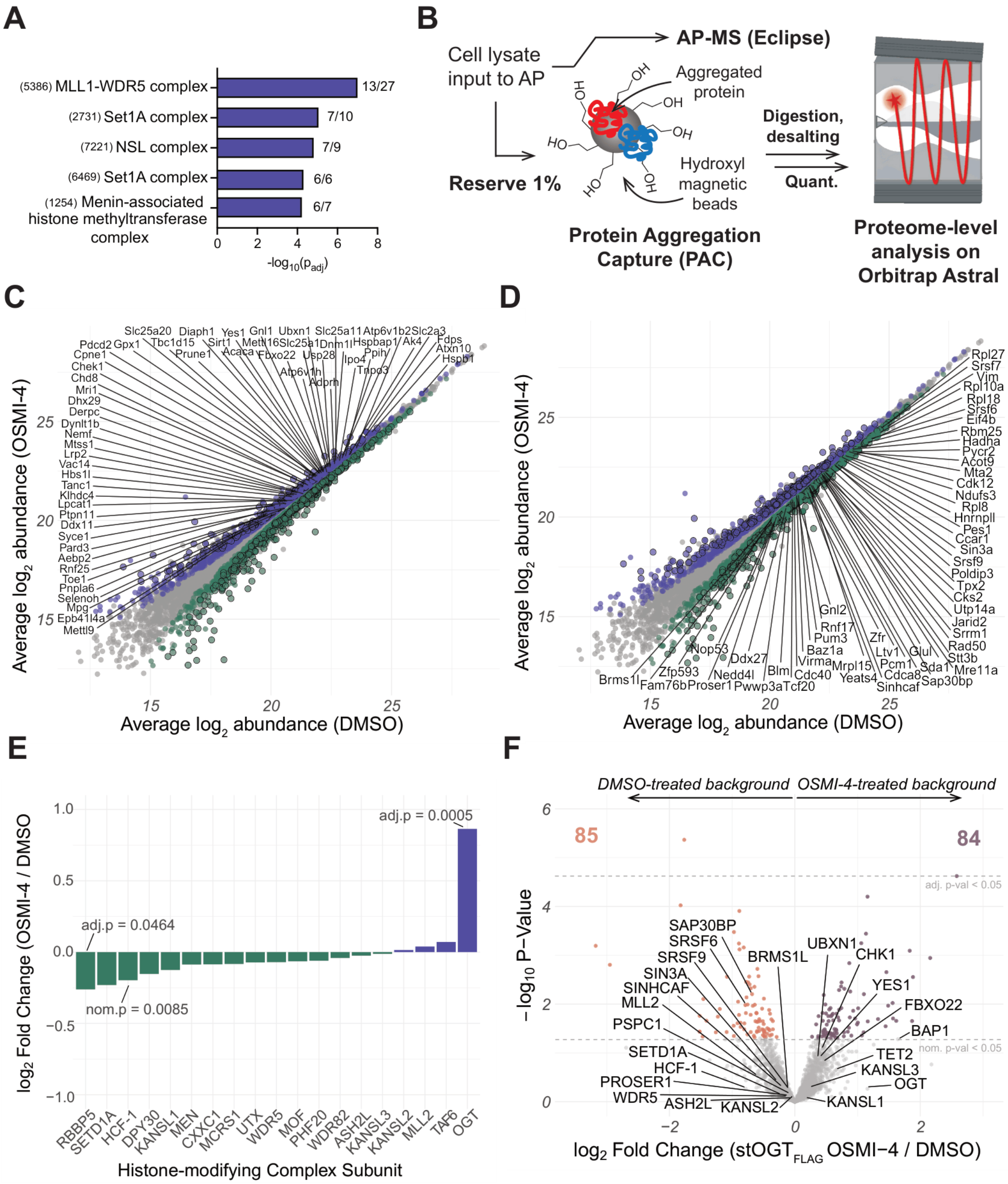

Figure S3A. Bar plot of CORUM complex terms relating to the NSL and COMPASS family complexes. All proteins that significantly increase in interaction with OGT were analyzed in g:Profiler with the following parameters changed from default: species = *Mus musculus*, FDR < 0.01, and no electronic GO annotations. Labels to the right of the bars indicate the number of proteins in the analyzed data set / number of proteins in the annotated CORUM complex. CORUM complex IDs are to the left of the complex name.

bioRxiv preprint doi: https://doi.org/10.64898/2026.09.01.748607; this version posted September 3, 2026. The copyright holder for this preprint (which was not certified by peer review) is the author/funder, who has granted bioRxiv a license to display the preprint in perpetuity. It is made available under a CC-BY 4.0 International license.

Figure S3B. Schematic of proteome-level sample analysis via LC-MS/MS. A portion (1%) of the sample input to the affinity purification was reserved as "proteome-level" samples. Protein aggregation capture (PAC) was used to remove contaminants (salts, detergent, cell debris) and allow for on-bead digestion. After MS sample preparation steps, samples were analyzed on an Orbitrap Astral using narrow window data-independent acquisition (nDIA).

Figure S3C. Scatter plot of proteins (56) that increase in interaction and abundance upon OSMI-4 treatment. The average $\log_2$ normalized protein abundance is plotted (OSMI-4 on y-axis; DMSO on x-axis). Larger dots with a black border are proteins that pass adjusted p-value < 0.05. Purple = interaction gain with OSMI-4; Green = interaction loss with OSMI-4; Gray = nominal $p > 0.05$. Proteins are labeled by their gene symbol.

Figure S3D. Scatter plot of proteins (55) that decrease in interaction and abundance upon OSMI-4 treatment. The average normalized protein abundance is plotted (OSMI-4 on y-axis; DMSO on x-axis). Larger dots with a black border are proteins that pass the threshold adj. p-value < 0.05. Purple = interaction gain with OSMI-4; Green = interaction loss with OSMI-4; Gray = nominal $p > 0.05$. Proteins are labeled by their gene symbol.

Figure S3E. Bar plot of $\log_2$ fold changes (OSMI-4 / DMSO) of NSL/COMPASS complex components. Values are from the OSMI-4- vs. DMSO-treatment proteome-level comparison. Proteins that pass a nominal or adjusted p-value threshold are labeled with their p-value.

Figure S3F. Volcano plot comparing OSMI-4- and DMSO-treated stOGT$_{FLAG}$ controls, an ancillary comparison as an example. Proteins enriched in the OSMI-4-treatment background are in mauve; proteins enriched in the DMSO-treatment background are in orange. Bolded values specify proteins passing the nominal p-value threshold $p < 0.05$.

bioRxiv preprint doi: https://doi.org/10.64898/2026.09.01.748607; this version posted September 3, 2026. The copyright holder for this preprint (which was not certified by peer review) is the author/funder, who has granted bioRxiv a license to display the preprint in perpetuity. It is made available under a CC-BY 4.0 International license.

Supplemental Figure 4.

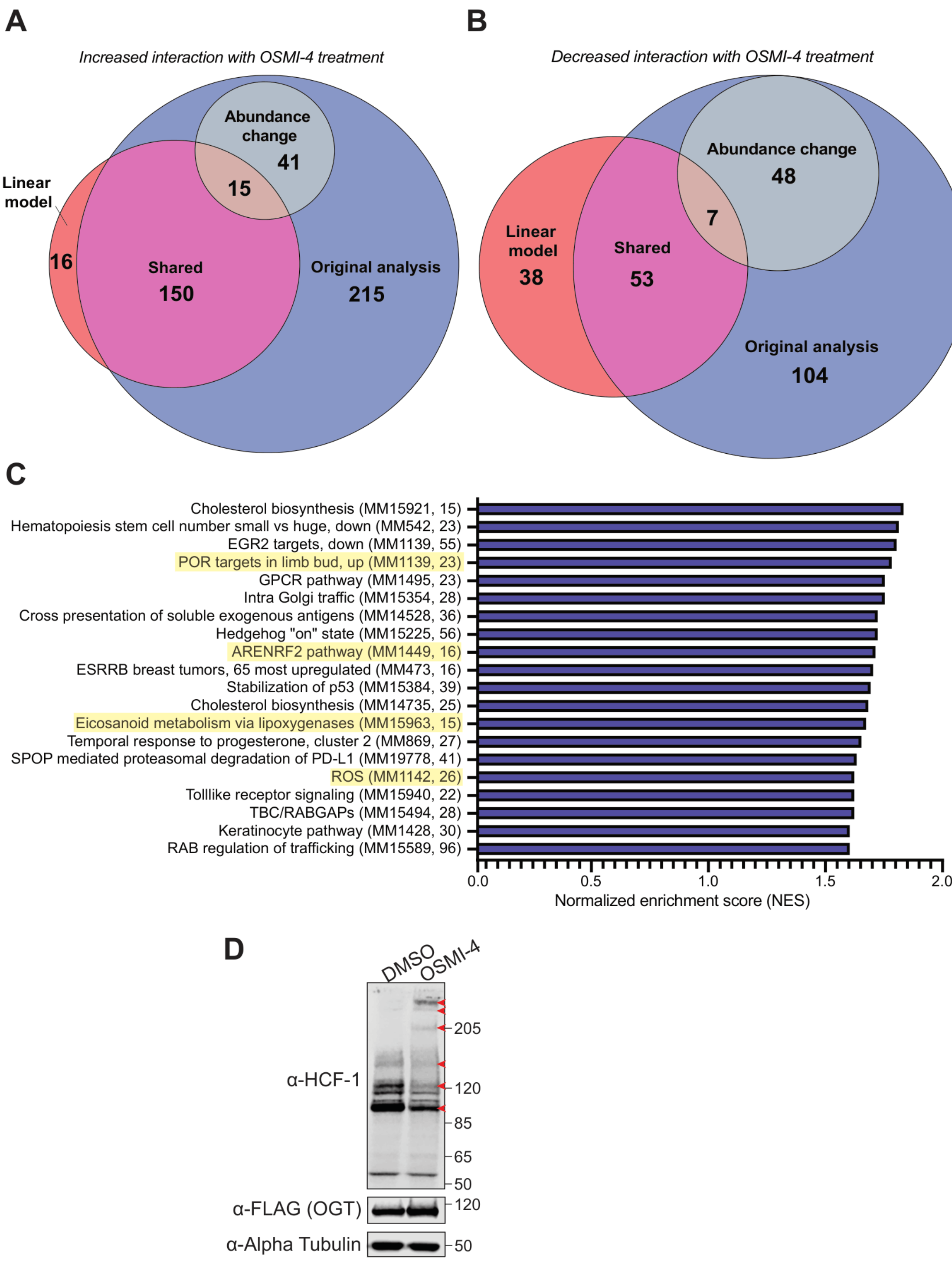

Figure S4A. Venn diagram comparing proteins identified by the original OSMI-4 vs. DMSO $fOGT_{FLAG}$ analysis (blue) and the model-fitted analysis (red) that increase in interaction with OGT upon OSMI-4 treatment. Significant interactors that are identified by both analyses are in pink. Proteins whose abundance increases commensurate with their interaction are in light blue.

bioRxiv preprint doi: https://doi.org/10.64898/2026.09.01.748607; this version posted September 3, 2026. The copyright holder for this preprint (which was not certified by peer review) is the author/funder, who has granted bioRxiv a license to display the preprint in perpetuity. It is made available under a CC-BY 4.0 International license.

Figure S4B. Venn diagram comparing proteins identified by the original OSMI-4 vs. DMSO fOGT$_{FLAG}$ analysis (blue) and the model-fitted analysis (red) that decrease in interaction with OGT upon OSMI-4 treatment. Significant interactors that are identified by both analyses are in pink. Proteins whose abundance decreases commensurate with their interaction are in light blue.

Figure S4C. Normalized enrichment scores (NES) of a GSEA comparing the OSMI-4 and DMSO-treated proteomes. The top 20 enriched gene set terms are shown. Yellow highlighted terms are related to oxidative stress or reactive oxygen species (ROS) production. The enrichment value used for GSEA analysis was $\log_2$ fold change. All gene sets pass a nominal p-value threshold $p < 0.05$. The parentheses next to the gene set name specify the mSigDB identifier and the number of genes (proteins) present in the gene set.

Figure S4D. Western blot analysis of HCF-1 cleavage patterns upon OSMI-4 treatment. Lysate was run on a 3-8% Tris-Acetate protein gel for SDS-PAGE prior to western blot. The HCF-1 antibody was raised specifically against the HCF-1 C-terminal half. Red arrowheads indicate cleavage products that either increase or decrease in abundance upon OSMI-4 treatment.

bioRxiv preprint doi: https://doi.org/10.64898/2026.09.01.748607; this version posted September 3, 2026. The copyright holder for this preprint (which was not certified by peer review) is the author/funder, who has granted bioRxiv a license to display the preprint in perpetuity. It is made available under a CC-BY 4.0 International license.

Supplementary Data Tables

Supplementary Table 1. nDIA proteomics analysis of 3x-FLAG-OGT and 2x-Strep-OGT mESC lines

Supplementary Table 2. AP-MS analysis comparing $fOGT_{FLAG}$, $fOGT_{ISO}$, and $stOGT_{FLAG}$ enrichments

Supplementary Table 3. AP-MS analysis of OSMI-4- vs. DMSO-treated $fOGT_{FLAG}$

Supplementary Table 4. nDIA proteomics analysis of 3x-FLAG-OGT mESC OSMI-4- vs. DMSO-treated proteomes

Supplementary Table 5. Linear model-fitted AP-MS analysis of OSMI-4- vs. DMSO-treated $fOGT_{FLAG}$ and $stOGT_{FLAG}$
